# Preoperative proteinuria correlates with renal function after partial nephrectomy for renal cell carcinoma

**DOI:** 10.1007/s00345-024-05042-w

**Published:** 2024-06-20

**Authors:** Michele Nicolazzini, Carlotta Palumbo, Francesca Porté, Gianmarco Bondonno, Paolo De Angelis, Maria Teresa Del Galdo, Alessandro Volpe

**Affiliations:** Division of Urology, Department of Translational Medicine, University of Eastern Piedmont, Maggiore Della Carità Hospital, Corso Mazzini 18, 28100 Novara, Italy

**Keywords:** Partial nephrectomy, Renal function, Chronic kidney disease, Proteinuria, Carcinoma, renal cell

## Abstract

**Purpose:**

Preoperative proteinuria is a prognostic factor of chronic kidney disease (CKD). We assessed the association between preoperative proteinuria and postoperative renal function after partial nephrectomy (PN).

**Methods:**

We retrospectively reviewed our records of patients with a single malignant renal mass who underwent PN between 2000 and 2021. Patients with data on preoperative proteinuria were included. Baseline characteristics and eGFR differences over time between patients with and without proteinuria were evaluated. Univariate and multivariable logistic regression models (LRM) tested for presence of CKDIII or higher at 12-month and at last follow-up.

**Results:**

Two hundred ninety-five patients were included. Twenty-two of them had preoperative proteinuria. No differences of age, smoking status, hypertension or diabetes, tumor size and use of ischemia were observed. Patients with proteinuria had a higher rate of CKD-III at baseline. At a median follow-up of 46.5 months (IQR 19–82), 117 patients developed de novo CKD-III, without differences in the two groups. No differences in decline in eGFR were observed. At univariate LRM, predictors of CKD-III at 12 months after PN were preoperative proteinuria (OR 3.2, 95%CI 1.4–7.8, p = 0.005), age and baseline eGFR, while predictors of CKD-III at last follow-up were age and baseline eGFR. At multivariable LRM, only baseline eGFR predicted CKD-III at 12-month and at last-follow-up.

**Conclusions:**

Preoperative eGFR is the only independent predictor of long-term renal function after PN. Preoperative proteinuria correlates with renal function at 12 months. Proteinuria should be assessed before PN to identify patients at higher risk of renal functional deterioration in the 12 months following PN.

## Introduction

Renal cell carcinoma incidence is increasing [1], especially in the elderly population. Surgery is the mainstay for treatment of localized RCC [2]. Nonetheless, the proportion of patients with baseline impairment of renal function, who are at higher risk of mortality, is increasing, too [3]. Thus, every effort should be made to preserve renal function, especially in this category of patients. Indeed, renal surgery has a well known impact on renal function, especially when radical nephrectomy is performed [4].

Therefore, it is important to predict the risk of development and/or progression of CKD after renal surgery. Many attempts have been done to identify predictors of CKD after nephrectomy [5]. Among other well-established risk factors such as advanced age, male gender and baseline eGFR, the role of proteinuria, defined as the presence of proteins in the urine [6], is still debated. In the medical literature, proteinuria is an established risk factor for progression to end stage renal disease (ESRD) and is significantly associated with cardiovascular disease and cardiovascular mortality [7]. However, to date, preoperative proteinuria has been demonstrated to be associated with a higher overall mortality after nephrectomy [8, 9], but there is no consensus on its role in predicting renal function after surgery, especially at long-term follow-up [10]. To address this unmet need, we investigated the association between preoperative proteinuria and renal functional outcomes after PN for RCC in a sizable single-center experience.

## Methods

We retrospectively reviewed our prospectively-maintained series of patients treated with PN for RCC. For the purpose of this study, only patients with available data on preoperative proteinuria and a minimum follow-up of 12 months were included. Cases with benign lesions at histology, surgery for multiple or recurrent renal masses and solitary kidneys were excluded.

Proteinuria was assessed with spot urine analysis performed during pre-operative assessment, usually one month before surgery. Proteinuria was considered significant in case of “ + or greater” in dipstick urine test, resulting in a > 30 mg/dl protein concentration in urine [6]. Pre- and post-operative renal function was assessed using serum creatinine (mg/dl) and eGFR according to the CDK-EPI equation (ml/min/1.73m^2^).

Follow-up was performed with a standardized protocol according to stage and grade at final pathology [11]. Outpatient visits were scheduled every six months for 3 years, then yearly up to 10 years. At each visit, laboratory examination including blood cell count, serum creatinine, electrolytes and urine examination were requested as well as abdominal imaging (ultrasound or contrast-enhanced CT scan) and chest imaging (X-ray or contrast-enhanced CT scan).

Descriptive statistics was performed using t-test and Mann–Whitney test to assess statistically significant differences in baseline characteristics and renal function between patients with and without preoperative proteinuria. Simple linear regression models (LRMs) assessed the differences in eGFR variation during follow-up between the two groups. Univariate and multivariable LRMs were used to evaluate predictors of stage III or higher CKD at 12-month and at last follow-up. The included variables were preoperative proteinuria (absent vs. present), preoperative eGFR, age, smoking status, personal history of diabetes and/or hypertension, tumor size, use of ischemia and ischemia time. All statistical tests were two-sided with a level of significance set at p < 0.05. Analyses were performed using the R software environment for statistical computing and graphics (version 4.2.0; http://www.r-project.org/).

## Results

From January 2000 to January 2022, 421 PNs were performed, with an increasing proportion over radical nephrectomy over time. Based on the inclusion criteria, 295 patients were eligible for the analysis. Twenty-two of them (7.4%) had preoperative proteinuria.

Baseline patients and tumor characteristics are shown in Table 1. No differences in terms of age, smoking status, hypertension or diabetes, tumor size, use of ischemia and ischemia time, as well as PN technique were observed. Additionally, patients with preoperative proteinuria had similar baseline eGFR of those without preoperative proteinuria (mean 70.1 ± 30.9 standard deviation [SD] vs 88.1 ± 19 SD respectively, p = 0.94). Conversely, the proportion of patients with preoperative CDK III or higher was significantly higher in patients with preoperative proteinuria (7 patients—31.8%—vs 14 patients—5.1%—respectively, p < 0.001).

**Table 1:** Baseline characteristics of 295 patients with and without preoperative proteinuria

Variable	Proteinuria		p
	Yes (n. 22, %)	No (n. 273, %)	
Gender, n. (%)			
Male	14 (63.6)	185 (67.8)	0.69
Female	8 (36.4)	88 (32.2)	
Age (years), median (IQR)	62 (54.7–71)	63 (54–71)	0.99
BMI, median (IQR)	27.1 (23.2–28.8)	26 (23.2–28.1)	0.99
Smoking history, n. (%)			
Yes/former	14 (63.6)	125 (45.8)	0.11
No	8 (36.4)	148 (54.2)	
Diabetes, n. (%)			
Yes	6 (27.3)	54 (19.8)	0.4
No	16 (72.7)	219 (80.2)	
Hypertension, n. (%)			
Yes	14 (63.6)	145 (53.1)	0.34
No	8 (36.4)	128 (46.9)	
CCI, n. (%)			
0	5 (22.7)	88 (32.2)	0.1
1–3	11 (50.0)	153 (56.0)	
> 3	6 (27.3)	32 (11.7)	
Side, n. (%)			
Right	12 (54.5)	148 (54.2)	0.97
Left	10 (45.5)	125 (45.8)	
Cystic feature, n. (%)			
Yes	3 (13.6)	30 (11.0)	0.7
No	19 (86.4)	243 (89.0)	
Mass size (mm), median (IQR)	37.0 (30.0–52.5)	33.0 (24.0–43.0)	0.7
Baseline eGFR, median (IQR)	83.3 (53.2–95.7)	91.0 (75.9–102.0)	0.7
CKD stage ≥ III, n. (%)			
Yes	7 (31.8)	14 (5.13)	< 0.001
No	15 (68.2)	259 (94.9)	
PADUA risk score, n. (%)			
Low (6–7)	6 (27.3)	110 (40.3)	0.25
Intermediate (8–9)	10 (45.5)	122 (44.7)	
High (> = 10)	6 (27.3)	41 (15.0)	
PN technique			
Open	8 (36.4)	54 (19.8)	0.17
Laparoscopic	7 (31.8)	98 (35.9)	
Robotic	7 (31.8)	121 (44.3)	
Use of ischemia, n. (%)			
Yes	16 (72.7)	228 (83.5)	0.19
No	6 (27.3)	45 (16.5)	
WIT (min), median (IQR)	18.0 (16.0–23.0)	18.0 (13.0–21.0)	0.8

*PN* partial nephrectomy, *WIT* warm ischemia time

**Fig. 1:**
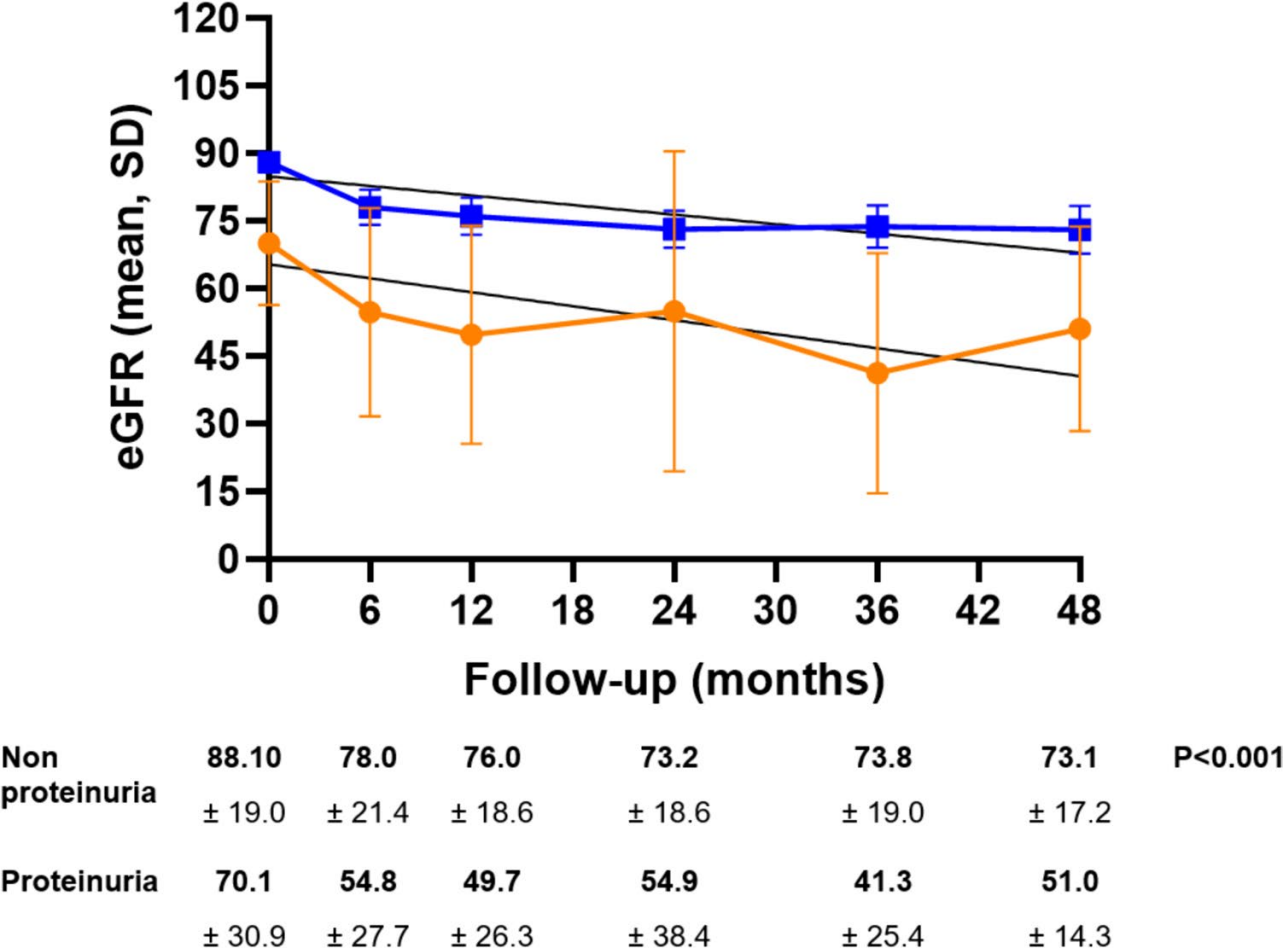
Mean (± SD) eGFR at each timepoint. Orange line represents patients with preoperative proteinuria, blue line represents patients without preoperative proteinuria. The two thin black lines are the results of linear regression model: *y* = *70.1 – 0.51* × for patients with preoperative proteinuria and *y* = *88 – 0.35* × for patients without preoperative proteinuria

The median follow-up was 46.5 months (interquartile range [IQR] 19–82). At each timepoint after PN (Fig. 1), patients with preoperative proteinuria had consistently lower mean eGFR values compared to those without proteinuria (p < 0.001). Nonetheless, LRMs did not show any statistically significant difference in renal function decline between patients with and without preoperative proteinuria (regression coefficient of − 0.51 and − 0.35 respectively, p = 0.6). Out of 274 patients with a normal baseline renal function, 117 patients developed de novo CKD III or higher after PN, without significant differences between patients with preoperative proteinuria (6 of 15, 40%) or without preoperative proteinuria (111 of 259, 42.8%) (p > 0.99).

At univariate LRMs predicting de novo CKD stage III or higher at 12 months (Table 2), presence of preoperative proteinuria (odds ratio [OR] 3.2, 95% confidence interval [CI] 1.4–7.8, p = 0.005), higher age (OR 1.05, 95% CI 1.02–1.07, p < 0.001) and lower baseline eGFR (OR 0.95, 95% CI 0.93–0.97, p < 0.001) were significantly associated with de novo CDK stage III or higher. At multivariable analyses, only lower baseline eGFR was an independent predictor of CKD stage III or higher (OR 0.95, 95%CI 0.94–0.97, p < 0.001).

**Table 2:** Univariate and multivariate logistic regression models predicting de novo chronic kidney disease stage III or higher

Variable	Univariate			Multivariate		
	OR	95% CI	p value	OR	95% CI	p value
De-novo CKD stage ≥ III at 12 months						
Preoperative proteinuria (yes)	3.28	1.41–7.78	0.006	1.34	0.47–3.73	0.57
Age (years)	1.05	1.03–1.08	< 0.001	1.02	0.98–1.05	0.24
Hypertension (yes)	1.53	0.94–2.51	0.08	1.06	0.58–1.93	0.85
Diabetes (yes)	1.52	0.77–2.95	0.21	0.88	0.38–1.94	0.76
eGFR (ml/min/1.73m^2^)	0.94	0.93–0.96	< 0.001	0.95	0.93–0.97	< 0.001
De-novo CKD stage ≥ III at last follow-up						
Preoperative proteinuria (yes)	2.31	0.98–5.28	0.051	0.73	0.24–2.12	0.57
Age (years)	1.06	1.03–1.08	< 0.001	1.01	0.98–1.05	0.35
Hypertension (yes)	2.04	1.21–3.37	0.005	1.66	0.89–3.08	0.11
Diabetes (yes)	1.37	0.68–2.68	0.35	0.62	0.26–1.41	0.26
eGFR (ml/min/1.73m^2^)	0.94	0.93–0.96	< 0.001	0.95	0.93–0.96	< 0.001

At univariate LRMs predicting de novo CKD stage III or higher at last follow-up (Table 2), older age (OR 1.05, 95%CI 1.03–1.08, p < 0.001) and lower baseline eGFR (OR 0.94, 95%CI 0.93–0.96, p < 0.001), but not presence of preoperative proteinuria (OR 2.3, 95%CI 0.97–5.3, p = 0.05) were significantly associated with de novo CKD stage III or higher. At multivariable analyses, only lower baseline eGFR was an independent predictor of de novo CKD stage III or higher (OR 0.94, 95%CI 0.92–0.96, p < 0.001).

## Discussion

Chronic kidney disease is one of the main cardiovascular risk factors and its prevalence is increasing especially in high-income countries [12]. When planning renal surgery for RCC, oncological safety should be balanced with preservation of renal function in order to minimize the risk of CKD progression and cardiovascular mortality [7]. While great efforts have been made to optimize preoperative assessment and surgical techniques with the aim to maximally preserve renal function, preoperative proteinuria is still underreported and its role in CKD development is still unclear. Therefore, we tested whether preoperative proteinuria is associated with renal function after PN for RCC. Our analysis results in several noteworthy findings.

First, patients with preoperative clinically significant proteinuria (i.e. > 30 mg/dL on spot urine analysis) represent a minority of those surgically treated and tend to have an already decreased baseline renal function. However, our data may underestimate the prevalence of proteinuria in patients with small renal masses, since in our experience a proportion of them are managed with non-surgical treatments, such as active surveillance or ablative therapies. Nonetheless, in patients with healthy kidneys, glomerular filtration and tubular excretion of proteins should be negligible. Therefore, the presence of detectable proteinuria should be considered as a marker of either pre-existing or subclinical kidney failure.

Second, there are no differences in the rate of renal functional decline nor in the development of de novo CKD stage III or higher between patients with and without preoperative proteinuria. These results are in contrast with expectations based on the role of preoperative proteinuria as a risk factor for CKD progression in the general population [7]. The effect of proteinuria may be underestimated in patients undergoing nephrectomy due to the presence of more impacting risk factors, like renal surgery itself [4]. Although not reaching statistical significancy, the curves representing eGFR variation over time (Fig. 1) in both groups suggest a faster decline during the first 6 months. This kinetics is consistent with that observed by Kim et al., that described an early decline in renal function seven days after PN, with a subsequent stabilization of the eGFR [13].

Third, in our analysis preoperative proteinuria results as a predictive factor of CKD at one year after PN. Previous studies had found similar results, summarized by Flammia et al. in a recent systematic review of the literature [10]. Zhang et al. described proteinuria as an independent predictor of loss of renal function stability after renal surgery, defined as a decline in eGFR > 50% or start of dialytic treatment [8]. Sun et al. identified presence of moderate or severe albuminuria as a predictive factor of CKD progression after both RN and PN [14]. Krane et al. found that preoperative proteinuria is a predictor of development of de novo stage III CKD after robot-assisted PN [15]. Tachibana et al. identified a correlation between presence of protein at dipstick urine analysis and presence of eGFR < 30 ml/min/1.73 m2 after PN in solitary kidney patients [16]. Bhindi et al. developed a predictive model of renal function after both RN and PN, including preoperative proteinuria in 24-h urine, together with other preoperative variables. This model, although needing external validation, underlines the role of preoperative proteinuria in predicting renal function after renal surgery [17]. To sum up, many authors agreed on the role of preoperative proteinuria in predicting loss in renal function after PN, but there is still no agreement on its role at long-term follow-up. Some of the already cited studies have a median follow-up of less than 2 years [15]. Kara et al. found in their series that preoperative proteinuria was associated with a higher risk of acute kidney injury and of decrease in renal function one month after PN, but did not observe any differences at 3 months and at last follow-up [18]. In our analysis we also found that at last follow-up only preoperative eGFR was an independent predictive factor of CKD stage III or higher. This may be explained by the high prevalence of impaired renal function at baseline in our patients with preoperative proteinuria: in these patients proteinuria can be seen as a sign of a more severe CKD rather than a marker of a latent preclinical kidney disease. Moreover, in previous literature definitions of the outcomes were not univocal, limiting generalization of results. Hence, in this study, we used a simple and broad definition of proteinuria in order to make our results reliable and reproducible.

Taken together, we demonstrated that preoperative proteinuria is a feasible and easily available marker for a higher risk of decreasing renal function after PN, especially in the first post-operative year. Hence, we suggest to include urine analysis in the preoperative work up of patients who are scheduled for renal surgery in order to allow a better preoperative planning and a tailored functional follow-up.

Despite its strengths, our study has limitations that should be acknowledged. The main limitation is the retrospective nature of the study. Second, proteinuria was assessed only as present vs. absent and therefore we were not able to assess whether different values of proteinuria may have had a different impact on postoperative renal function. Third, since most patients with proteinuria had a worse baseline renal function, the strength of its association with postoperative renal impairment may have been decreased.

## Conclusion

The presence of preoperative proteinuria at spot urine analysis significantly correlates with renal function 12 months after PN, but not at long-term follow-up. Preoperative eGFR remains the main predictor of long-term renal function after PN. Nonetheless, proteinuria is easily assessable before PN and may help identifying patients that may be at higher risk of renal functional deterioration after surgery. This may have an impact on both surgical approach and postoperative care, aiming at further minimizing potential risks for renal damage.

## Data Availability

The data that support the findings of this study are not openly available due to reasons of sensitivity and are available from the corresponding author upon reasonable request.
